# The Role of Transforming Growth Factor-β (TGF-β) in Asthma and Chronic Obstructive Pulmonary Disease (COPD)

**DOI:** 10.3390/cells13151271

**Published:** 2024-07-29

**Authors:** Krzysztof Kraik, Maciej Tota, Julia Laska, Julia Łacwik, Łukasz Paździerz, Łukasz Sędek, Krzysztof Gomułka

**Affiliations:** 1 Student Scientific Group of Internal Medicine and Allergology, Clinical Department of Allergology and Internal Diseases, Institute of Internal Diseases, Wroclaw Medical University, 50-369 Wrocław, Poland; 2 Student Scientific Group of Microbiology and Immunology, Department of Microbiology and Immunology, Zabrze, Medical University of Silesia in Katowice, 40-055 Katowice, Poland; 3 Department of Microbiology and Immunology, Zabrze, Medical University of Silesia in Katowice, 40-055 Katowice, Poland; 4 Clinical Department of Allergology and Internal Diseases, Institute of Internal Diseases, Wroclaw Medical University, 50-369 Wrocław, Poland

**Keywords:** asthma, COPD, pathogenesis, TGF-beta, transforming growth factor, chronic obstructive pulmonary disease, airway remodeling, pulmonary

## Abstract

Asthma and chronic obstructive pulmonary disease (COPD) represent chronic inflammatory respiratory disorders that, despite having distinct pathophysiological underpinnings, both feature airflow obstruction and respiratory symptoms. A critical component in the pathogenesis of each condition is the transforming growth factor-β (TGF-β), a multifunctional cytokine that exerts varying influences across these diseases. In asthma, TGF-β is significantly involved in airway remodeling, a key aspect marked by subepithelial fibrosis, hypertrophy of the smooth muscle, enhanced mucus production, and suppression of emphysema development. The cytokine facilitates collagen deposition and the proliferation of fibroblasts, which are crucial in the structural modifications within the airways. In contrast, the role of TGF-β in COPD is more ambiguous. It initially acts as a protective agent, fostering tissue repair and curbing inflammation. However, prolonged exposure to environmental factors such as cigarette smoke causes TGF-β signaling malfunction. Such dysregulation leads to abnormal tissue remodeling, marked by excessive collagen deposition, enlargement of airspaces, and, thus, accelerated development of emphysema. Additionally, TGF-β facilitates the epithelial-to-mesenchymal transition (EMT), a process contributing to the phenotypic alterations observed in COPD. A thorough comprehension of the multifaceted role of TGF-β in asthma and COPD is imperative for elaborating precise therapeutic interventions. We review several promising approaches that alter TGF-β signaling. Nevertheless, additional studies are essential to delineate further the specific mechanisms of TGF-β dysregulation and its potential therapeutic impacts in these chronic respiratory diseases.

## 1. Introduction

The TGF-β superfamily comprises various isoforms of TGF-β (TGF-β1, TGF-β2, and TGF-β3), along with activins, nodals, bone morphogenetic proteins (BMPs), growth and differentiation factors (GDFs), and Müllerian-inhibiting substance (MIS) [1]. Transforming growth factor-beta (TGF-β) is a pleiotropic cytokine that governs an extensive array of cellular functions, encompassing cell growth, apoptosis, differentiation, migration, and extracellular matrix production [1].

The active form of TGF-β is constituted by a 25 kDa dimer of two polypeptides interconnected by a disulfide bond and hydrophobic interactions [2]. TGF-β can be transformed to its active form by thrombospondin 1 (TSP-1), reactive oxygen species (ROS), acids, bases, proteases, and integrins. The latter are noteworthy, as they facilitate the activation of TGF-β by inducing its dissociation from the inactive complex [3,4]. Three TGF-β ligands (TGF-β1, 2, and 3) exhibit varying degrees of affinity towards three isoforms of the TGF-β receptors (TGF-β R). TGF-β RI and TGF-β RII are both tyrosine and serine/threonine kinases, while TGF-β RIII lacks kinase activity [5].

In physiological conditions, TGF-β signaling is essential for many biological processes, including embryonic development, wound healing, tissue repair, cell proliferation, migration, apoptosis, tissue homeostasis, and immune homeostasis [6,7]. The TGF-β plays a pivotal role throughout embryonic development in regulating cellular differentiation, facilitating epithelial and endothelial to mesenchymal transitions (EMT/EndMT), and orchestrating apoptosis. These functions are critical for ensuring appropriate histogenesis and organogenesis. Moreover, TGF-β facilitates wound healing through its involvement in several critical phases, including inflammation, re-epithelialization, angiogenesis, and activation of fibroblasts [4].

Numerous studies have demonstrated the role of TGF-β in the development and progression of various diseases, including chronic kidney disease (CKD), neoplastic diseases, idiopathic pulmonary fibrosis (IPF), cystic fibrosis (CF), IgA nephropathy, asthma, and COPD [4,8,9]. TGF-β is considered one of the most potent profibrotic cytokines, and extensive research has revealed that its overexpression is a common feature in most fibrotic diseases [10,11].

### 1.1. Canonical (Smad) Pathway

**Figure 1:**
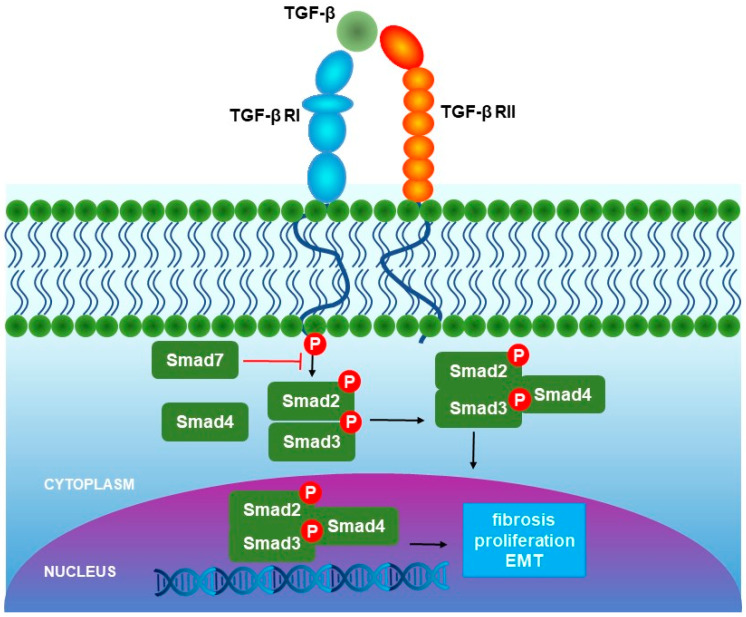
Canonical (Smad) pathway of TGF-β signaling.

The canonical pathway (activin/TGF-β activated) begins with TGF-β binding to the TGF-β RII, which recruits and phosphorylates TGF-β RI (ALK5). Thereupon, phosphorylated TGF-β RI recruits Smad4 and translocates to the nucleus, where it controls the transcription of TGF-β target genes (Figure 1) [5]. Eight Smad proteins exist in mammals, which can be categorized into three distinct classes according to their functional roles: the receptor-activated Smads (R-Smads), which include Smad1, 2, 3, 5, and 8; the common mediator Smad (Co-Smad), represented solely by Smad4; and the inhibitory Smads (I-Smads), comprising Smad6 and 7 [1]. TGF-β1 operates through a highly defined canonical signaling cascade that triggers the phosphorylation and activation of Smad2 as well as Smad3 in the TGF-β RI and Smad4 incorporation, which allows the complex to translocate to the nucleus and control the transcription of specific genes [12].

### 1.2. Non-Canonical (Non-Smad) Pathway

Non-canonical TGF-β signaling pathways, which transduce without interaction with Smad proteins, utilize a variety of kinases, including p38, AKT, and ERK1/2 [13]. A non-canonical pathway is known to participate in the pathogenesis of fibrosis [3]. In vitro analyses demonstrate that Smad-mediated signaling and TGF-β-induced non-Smad pathways frequently exhibit interconnectivity. Smad signaling is characterized by extensive interactions with various non-Smad pathways that are essential in regulating fibrotic responses, including MAPKs, the Wnt/β-catenin axis, phosphoinositide 3-kinase (PI3K)/protein kinase B (AKT) pathway (PI3K-AKT), Janus kinase 2/signal transducer and activator of transcription 3 (JAK2/STAT3), nuclear factor kappa B (NF-κB), Rho GTPases, and Notch cascades [14,15,16,17,18,19]. However, the in vivo relevance of these interactions between Smad-dependent and non-Smad pathways in facilitating the fibrogenic effects of TGF-β is not well elucidated and remains an area requiring further investigation.

### 1.3. Aims of the Study

The current study aims to review the scientific literature on the role of TGF-β in asthma and COPD. A comparison between these two pulmonary obstructive diseases is presented. Moreover, we aim to find gaps and inconsistencies in the current state of knowledge and propose further research directions on this topic. Furthermore, we aim to organize the results of previous research and present them clearly. To date numerous studies have been published, although their methodology is divergent and includes both studies on humans and animals. Moreover, this review provides a comprehensive overview of the various therapeutic strategies targeting the components of the TGF-β signaling pathway. Finally, we explore the future perspectives in this area.

## 2. The Role of TGF-β in Chronic Obstructive Pulmonary Disease (COPD)

Chronic obstructive pulmonary disease (COPD) is a respiratory disease that accounts for most of the deaths from chronic lower respiratory diseases. In 2019, it was the third leading cause of death globally and in the USA and the fifth in European WHO Region [20]. Numerous studies indicate the involvement of TGF-β in the pathogenesis of COPD [21,22,23].

### 2.1. The Levels of TGF-β in COPD

Many researchers investigated the level of TGF-β in COPD patients. In a study conducted on smokers and former smokers, people with COPD had approximately twice the level of TGF-β1 mRNA and TGF-β_1_ proteins in the bronchial and alveolar epithelium than smokers and ex-smokers who did not have COPD [24]. Moreover, the amount of TGF-β in the induced sputum in people with COPD was higher than in healthy people, including people who were smoking regularly [25]. Another study found a correlation between the amount of TGF-β in cells present in the sputum cells and the severity of obstruction of the airways. Moreover, it confirmed its increased level in COPD patients [26]. Increased TGF-β levels were also revealed in smokers’ basal reticular membrane of the lungs, and the increase was especially well-marked in COPD patients [27]. An increase in TGF-β levels in COPD patients was also observed. TGF-β levels correlated negatively with forced expiratory volume in the first second (FEV_1_) and forced vital capacity (FVC) [22]. In another study, increased TGF-β concentration in serum was found in COPD patients and was associated with lung function in the GOLD scale [28]. Moreover, in the study comparing the level of TGF-β1 in 59 smoking and 66 non-smoking pregnant women, there was a significantly higher concentration of TGF-β1 in blood of smoking women [29]. On the contrary, Kokturk et al. found no significant differences in TGF-β1 expression in bronchial biopsies between COPD and healthy groups. However, their study included only 13 COPD and 10 healthy participants [30].

### 2.2. Genetic Background of TGF-β and COPD Association

Moreover, the presence of the C allele in the TGF-β-related single nucleotide polymorphism (SNP) rs1982073 detected in Caucasians was related to reduced risk of COPD; however, this relationship was not confirmed among people of the Mongolian race [31]. Also, the C509T and T869C polymorphisms detected among the Chinese population were not related to the risk of COPD [22].

### 2.3. TGF-β Takes Part in the Development of Emphysema in COPD

**Figure 2:**
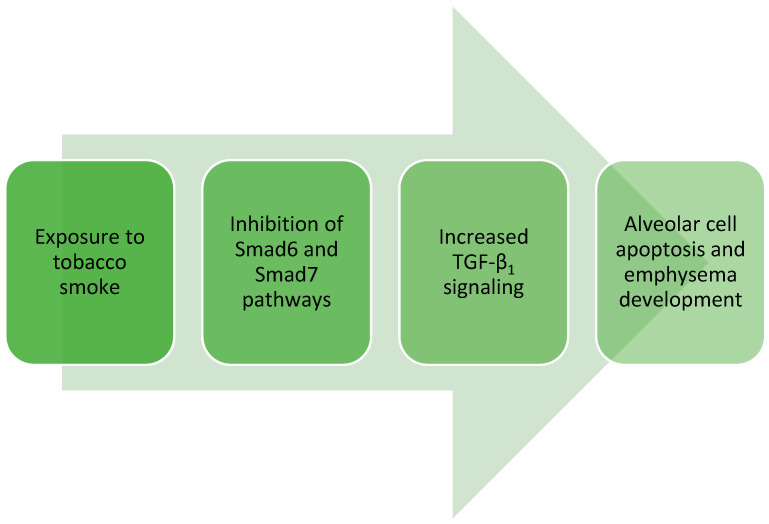
The effect of exposure to tobacco smoke on emphysema development.

TGF-β contributes to the thickening of the walls of small alveoli and their fibrosis, revealed by studies performed on a mouse model. Tobacco smoke inhibits the Smad6 and Smad7 pathways, which inhibit TGF-β1 signaling. As a result, an enhancement in TGF-β1 signaling was observed, which may contribute to alveolar cell apoptosis and the development of massive emphysema (Figure 2). However, other studies indicate that also TGF-β1 deficiency contributes to the development of emphysema [32].

Moreover, TGF-β stimulates pulmonary macrophages to produce matrix metalloproteinases (MMPs) in mice. Overproduction of these enzymes damage the alveoli and causes emphysema [33]. Other studies reveal that TGF-β may affect the activity of some enzymes. Increased TGF-β1 levels decreased the expression of SLPI (protease inhibitor) in rats, thereby exposing adjacent tissues to damage [34].

On the contrary, in another mouse model, the loss of TGF-β RII receptors resulted in an increased prevalence of emphysema, which is one of the components of COPD. Hence, this finding may indicate the role of TGF-β in preventing emphysema [35]. In another study, the level of TGF-β1 was measured in the lung tissue of COPD patients and non-smokers. The TGF-β1 levels were lower in COPD patients than in non-smokers, suggesting that Smad2/3 and Smad7 pathways, responsible for COPD development, also depend on other factors [36].

### 2.4. Protective Role of Club Cells

In a recent study, Tian et al. revealed in a mice model study that inhibiting the TGF-β1/ALK5 pathway may affect the MEK/ERK pathway and slow down the development of COPD by improving the function of club cells [37]. Function of club cells might be significant in understanding pathophysiology of COPD due to reduction of club cells and their products in smoking patients at risk of developing COPD, which was shown in many studies. Club cells secretory products serve many functions in airways such as reducing oxidative stress and inflammation or protection from xenobiotics. Secretoglobin family 1A member 1 (SCGB1A1) reduces inflammation caused by microbial or environmental factors due to inhibition of interferon gamma (INF-γ) and phospholipase A2 [38,39].

### 2.5. The Role of TGF-β in Airway Remodeling in COPD

TGF-β1 takes part in airway remodeling in COPD. Exposure to tobacco smoke, which is an essential factor that is almost always present in the history of COPD patients, increases the production of TGF-β1 in the respiratory tract. TGF-β1 causes an increase in the production of extracellular matrix, proliferation of smooth muscle cells, and a change in the phenotype of epithelial cells to mesenchymal [40]. The function of TGF-β, produced by airway epithelial cells and macrophages, is also to enhance the proliferation of fibroblasts and to accelerate fibrosis, which contributes to the unfavorable bronchial remodeling process [41]. Airway smooth muscle cells may also have a role in producing extracellular matrix and fibrotic changes [42].

TGF-β also is responsible for inhibition of mucus secretion in the airways. It was revealed that smokers with COPD had reduced expression of TGF-β RII than smokers not suffering from COPD. Due to this change, the signaling of TGF-β1 is reduced and excessive amounts of mucus are secreted into the airways. Moreover, it was found that the larger the bronchial glands in COPD are, the lower is the expression of TGF-β RII. The authors of the cited study claim that another theory, which assumes internalization of TGF-β RII due to TGF-β overexpression, is unlikely due to the low dynamics of this process [43]. Furthermore, there are other factors influencing mucus production, including interleukin-13 (IL-13) and epidermal growth factor receptor (EGFR). It is has been found that IL-13 increases the expression of calcium-activated chloride channel-1 (CLCA1), which activates MAPK13, which further induces mucin production [44]. Although TGF- α is a ligand of EGFR, which increases mucus secretion in the airways, no studies found the interactions between EGFR and TGF-β. In addition, TGF-β, directly or indirectly, affects angiogenesis in COPD-affected lungs, e.g., by inducing VEGF secretion [27].

The increased expression of TGF-β1 in the pulmonary alveoli and respiratory tract observed in COPD patients has been recognized as one, although not the only, factor influencing the excessive influx of macrophages and mast cells into these tissues. Increased expression of TGF-β receptors (both TGF-β RI and TGF-β RII) was also observed among macrophages from COPD patients in comparison to macrophages of healthy people, suggesting the role of TGF-β as a factor involved in macrophage recruitment. The influx of macrophages may be caused directly by the presence of TGF-β or by the induction of monocyte chemoattractant protein (MCP-1) secretion in the presence of TGF-β [24]. However, another study revealed reduced TGF-β1 secretion by macrophages of COPD patients, suggesting an anti-inflammatory function of TGF-β [45].

Furthermore, TGF-β takes part in macrophage polarization via the TGF-β/Smad pathway. In the presence of bone morphogenic protein (BMP) and activin-binding membrane inhibitor (BAMBI), the TGF-β/Smad pathway is inhibited, and macrophages are polarized towards the M1 phenotype. Concomitantly, the number of M2 macrophages is decreased, which causes increased differentiation of T-cells towards Treg lymphocytes (Tregs). As a result, Th17/Treg lymphocyte ratio is disrupted, leading to increased inflammation [46]. In COPD, BAMBI is overexpressed, which results in the previously mentioned Th17/Treg imbalance and increased inflammation. TGF-β probably causes the overexpression of BAMBI, thereby reducing its anti-inflammatory function of promoting the differentiation of T-cells towards Tregs. Moreover, exposure to tobacco smoke may also cause excessive production of BAMBI [47]. However, some studies reveal that there may be another mechanism, which is independent of TGF-β, that causes an increase in the levels of BAMBI [48].

**Figure 3:**
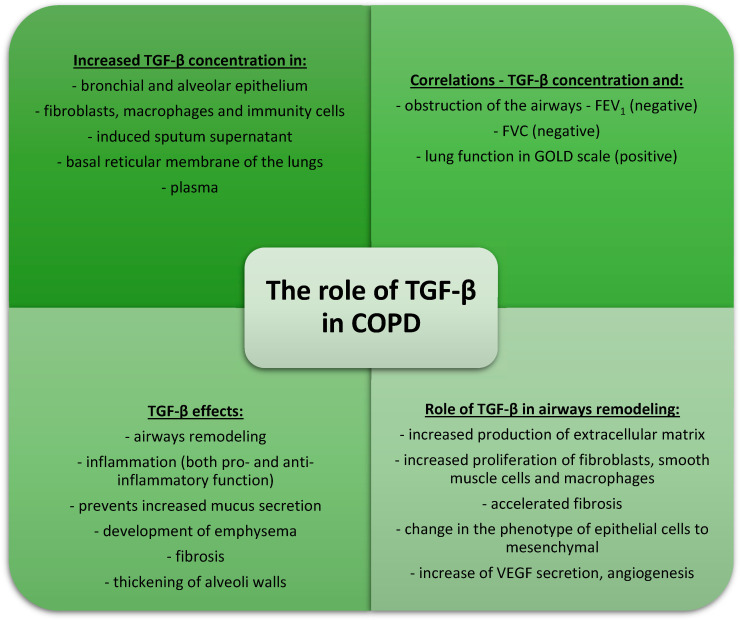
The role of TGF-β in COPD.

In conclusion, TGF-β plays a role in the development of emphysema and airway remodeling in COPD. Its role in the induction of inflammation is unclear, although most authors emphasize the anti-inflammatory role of TGF-β. However, most of the data come from studies performed on mouse models, which do not necessarily elucidate the role of TGF-β in humans. The information about the role of TGF-β in COPD is summarized in Figure 3.

## 3. The Role of TGF-β in Asthma Pathogenesis

Asthma is the most common chronic disease among children. In 2019, it was estimated that 262 million people were affected by asthma, which constituted approximately 3.4% of the total population [49].

### 3.1. The Levels of TGF-β in Asthma

The role of TGF-β in asthma was described earlier in 2002 by Duvernelle et al. Excessive expression of TGF-β1 mRNA was described in a population of asthmatics with moderate and high disease severity. The most involved cell types in asthma are inflammatory cells, especially activated eosinophils. It is relevant that TGF-β has a dual function—it can be both a pro-inflammatory cytokine (participating in chemotaxis, proliferation, activation, differentiation, and viability of inflammatory cells and in the release of other pro-inflammatory cytokines and reactive oxygen species by cells building the respiratory tract) and anti-inflammatory (inhibiting the proliferation of T and B lymphocytes and participating in the suppression of Th2 lymphocytes and the production of other cytokines). Studies in mice confirmed this hypothesis—mice with hypoexpression and overexpression of TGF-β showed similar characteristics of inflammation. The role of TGF-β in remodeling the respiratory tract was also noticed and was determined to be dominant in this process compared to platelet-derived growth factor (PDGF) and insulin-like growth factor (IGF-1). The involvement of TGF-β has been associated mainly with fibrotic processes in the lungs and thickening of the basement membrane [50]. It is known that the pro- or anti-inflammatory role of TGF-β may depend on the microenvironment and cellular conditions [51].

There are many studies investigating the concentration of TGF-β in the airways and plasma of asthma patients. Higher plasma TGF-β concentrations were found in patients with asthma in both children and adults, as compared to healthy persons [52,53]. Moreover, children with severe asthma have a higher concentration of TGF-β1 in bronchoalveolar lavage fluid than children with mild or moderate asthma [54]. The increased concentration of TGF-β1 was also found in the submucosa of large airways of asthmatics when compared to healthy individuals [30] and in induced sputum supernatants in children with moderate or intermittent asthma compared to healthy children [55]. All these studies indicate increased levels of TGF-β in the airways of asthmatics.

In a study by Keskin et al., no differences in TGF-β1 concentration were observed in exhaled air in asthmatic children compared to healthy ones. However, after the exercise challenge test, the exhaled TGF-β1 concentration was significantly higher in asthmatic children without exercise-induced bronchospasm than in asthmatic children with exercise-induced bronchospasm. The exercise challenge test did not significantly affect the concentration of TGF-β1 in healthy children. Furthermore, the concentration of exhaled TGF-β1 was associated significantly with FEV_1_ and asthma control test scores. Moreover, the concentration of exhaled TGF-β1 was significantly lower in asthmatic children with exacerbation than in children with stable asthma. These findings suggest that TGF-β1 may have a protective role in airway hyperreactivity and protects against asthma exacerbations [56].

Reduced expression of TGF-β receptors (TGF-β RI and TGF-β RII) was also found in asthmatics, including a reduction in TGF-β RI in patients with severe asthma [57] and a reduction in TGF-β RII in children with asthma (in comparison to healthy children and children with other atopic diseases) [58]. It may be caused by the process of receptor downregulation, which would indicate the chronic or massive exposition of cells to TGF-β.

### 3.2. Genetic Background of TGF-β and Asthma Association

The role of TGF-β in the pathogenesis of asthma is also indicated by a study performed on the Chinese population, which linked a single nucleotide polymorphism (SNP) of the TGF-β1 promoter (rs1800469 identical to C509T) with susceptibility to asthma development and revealed a possible association between another SNP (rs2241712) of the TGF-β1 promoter and this susceptibility [59]. The C509T SNP was also identified as a potential risk factor for the development of asthma in a meta-analysis involving the Chinese population [60]. In another study, the C509T polymorphism was associated with increased airflow obstruction and reduced eosinophilic inflammation [61]. C509T has been associated with excessive TGF-β1 transcriptional activity. The T869C SNP has also been associated with asthma. The T869C SNP is a part of the TGF-β1 gene, associated with excessive amounts of TGF-β1 mRNA and TGF-β1 production in peripheral cells [62]. Other authors also claimed that C509T and T869C polymorphisms are connected to asthma susceptibility [63,64]. Additionally, a recent study in the Polish population showed that the rs8109627 SNP in the TGF-β1 gene is more commonly found in controlled asthma, and the rs2796822 SNP in the TGF-β2 gene is more commonly found in uncontrolled asthma [65]. Another recent Polish study revealed that rs10779329 and rs4903359 SNPs of the TGF-β2 gene are associated with an increased risk of asthma development [66].

### 3.3. The Role of TGF-β in Asthma

Airway epithelial cells and eosinophils are the primary sources of TGF-β1 in asthma. TGF-β1 contributes to the hyperactivity of bronchial smooth muscles and the remodeling of the respiratory tract. TGF-β activates the expression of the Foxp3 gene in naive T lymphocytes, which, with simultaneous stimulation of the TCR, causes the differentiation of these cells into Treg lymphocytes, which inhibit inflammation and secrete TGF-β. Moreover, TGF-β inhibits the differentiation of Th1 and Th2 lymphocytes. However, it also activates Th17 lymphocytes, which can activate Th2 lymphocytes, responsible for the exacerbation of inflammation. There are also studies suggesting the involvement of TGF-β in the maturation of CD8+ T-cells and NKT lymphocytes. Hence, those studies show both the pro- and anti-inflammatory role of TGF-β in asthma [35,67]. Other researchers claim that overexpression of TGF-β1 in Th2 lymphocytes reduces bronchitis and airway hyperreactivity, and TGF-β1 derived from eosinophils increases hyperreactivity and contributes to a more severe course of asthma. However, the role of macrophage-derived TGF-β1 has not yet been determined [67,68]. Furthermore, TGF-β inhibits the activity of nuclear factor erythroid-2 (Nrf2), a factor responsible for the regulation of glutathione, by increasing the expression of activating expression factor-3 (ATF-3) mRNA. ATF-3 overexpression leads to displacement of Nrf2 coactivator, and, as a result, it inhibits the transcription requiring Nrf2 presence. Through this process TGF-β1 contributes to increases in oxidative stress [69].

It is also worth noting that excessive expression of TGF-β2 has been found in asthmatic patients [57], which can be associated with excessive mucus secretion in the respiratory tract [51,70].

### 3.4. The Role of TGF-β in Airway Remodeling in Asthma

Airway remodeling is a clinically significant, irreversible complication of asthma. If asthma is not treated correctly, the airways are constantly damaged and repaired, which results in irreversible exacerbation of obstruction due to thickening of the airway walls, loss of elasticity of the bronchi, and narrowing of the airways. Many studies have revealed the involvement of TGF-β in this process [51,52,67,68,71,72,73,74,75].

Airway remodeling includes many processes in which TGF-β is involved. These include alterations in the airway epithelium, peribronchial fibrosis, an increase in bronchial smooth muscle mass, goblet cell hyperplasia, and changes in the airway microvasculature [71]. These changes may also occur in mild asthma, in which thickening of the basement membrane has been found in children [58]. TGF-β, which is involved in these processes, is secreted not only by inflammatory cells but may also be secreted by fibroblasts and bronchial smooth muscle cells [73].

TGF-β affects airway epithelial cells through multiple signaling pathways. Without additional exposure to inflammatory mediators, harmful factors, and allergens, TGF-β acts through the Smad2/3 pathways, which is associated with anti-apoptotic effects and the risk of hypertrophy. In case of exposure to the above mentioned factors, the pro-apoptotic mitogen-activated protein kinase (MAPK) pathway is activated as a response to TGF-β. Smad2/3 pathways are also inactivated by the Smad7 pathway. This results in the exfoliation of the respiratory epithelium, which, together with repair processes disorder, contributes to the remodeling of the tissues underneath. In the presence of TGF-β, the Fas-related apoptosis pathway may also be stimulated in the alveoli and inhibited in the larger bronchi. Moreover, the presence of TRAIL factor increases the expression of TGF-β1 [51,67,71,74].

Remodeling of peribronchial tissues includes thickening of the basement membrane or fibrosis. These changes occur due to the deposition of the extracellular matrix, which includes collagen type I, collagen type III, and fibronectin, by fibroblasts and myofibroblasts. TGF-β enhances the proliferation and differentiation of these cells (at low levels of TGF-β), prevents their apoptosis, and takes part in the release of IL-6 and CTGF (connective tissue growth factor), which enhance the production of extracellular matrix and tissue inhibitors of metalloproteinases (TIMP), which inhibit collagenases. TGF-β can also reduce lung elasticity by activating the Smad7 pathway, which controls the synthesis of decorin, which is involved in the organization of collagen fibers, among others. The more dense the collagen arrangement, the less elastic are the airways [51,52,67,71,72,73,74]. This causes greater stiffness of the airways, which prevents effective bronchodilation and results in obstruction. Moreover, TGF-β1 may induce the production of extracellular matrix by airway smooth muscle cells, which results in fibrosis-like changes [42].

It was also found that the role of TGF-β1 may be modulated by secreted modular calcium-binding protein 2 (SMOC2). SMOC2 enhances the role of TGF-β1 as a factor promoting the proliferation and migration of fibroblasts as well as fibroblasts transformation to myofibroblasts (FMT) [76]. A study on an asthmatic rats model revealed similar results [77]. Another study suggests that FMT depends on TGF-β/Smad1/5/(8)9 pathway impairment. It was found that asthmatic patients had increased activity of the profibrotic TGF-β/Smad2/3 pathway and reduced activity of the antifibrotic TGF-β/Smad1/5/(8)9 pathway, and fibrotic changes occurred due to this imbalance [78].

Currently, available studies are contradictory regarding the effect of TGF-β on the production of MMPs, including collagenases. Some researchers claim that TGF-β stimulates MMPs production, while other authors report an opposite role of TGF-β [71,74].

Another process involved in the remodeling of airways is the increase in the mass of airway smooth muscles. TGF-β, secreted by muscle cells in low concentration, leads to the proliferation of smooth muscle cells through the MAPK pathway and the α5β1 receptor. The involvement of the Smad3 pathway in this process was also demonstrated in a mouse model, with an observation that high concentrations of TGF-β inhibited this process. TGF-β also has an anti-apoptotic effect on smooth muscle cells and causes the migration of these cells towards the epithelium by controlling the expression of MMPs and TIMPs in these cells [51,67,71,72].

There are also reports that TGF-β2 increases mucus secretion in the respiratory tract through stimulating the processes of transcription and translation of mucin. TGF-β2 also causes proliferation and hyperplasia of goblet cells in the bronchi [51,67,71]. An increased amount of mucus and the growth of goblet cells contribute to the severity of obstruction [67]. Moreover, TGF-β2 is one of the mediators through which IL-13 increases mucus secretion. Subsequently, TGF-β2 increases the expression of IL-6, which stimulates the goblet cells [51]. IL-13 also induces secretion of activin A, which belongs to TGF-β superfamily, which also is hypothesized to increase mucus secretion. It is hypothesized that activin A may regulate the production of IL-13 and due to this, indirectly increases mucus secretion [79]. On the contrary, in an in vitro study, it was found that adding TGF-β1 to human bronchial epithelial (HBE) cell culture did not affect mucins production, while adding TGF-β2 decreased it. Furthermore, adding TGF-β2 to cells, which were induced to produce mucins by addition of IL-13, resulted in a reduction in IL-13′s effect [80]. Due to these contradictions, the effect of TGF-β2 on mucus secretion in asthma is uncertain.

The effect of TGF-β on changes in bronchial microcirculation depends on the balance between the secretion of the factors that stimulate (e.g., VEGF and the Smad3 pathway protein) or inhibit the vascular growth or even cause apoptosis of vascular endothelial cells (TGF-β itself) [51,71].

### 3.5. The Interactions of Corticosteroids on TGF-β Expression in Asthma

A significant gap in current knowledge is the lack of consistent information about the effect of corticosteroids on TGF-β expression. Some researchers found no effect of corticosteroids on TGF-β expression and airway remodeling in patients with moderate to severe asthma [71]. Meanwhile, other researchers observed that corticosteroids reduced the expression of TGF-β and inhibited unfavorable changes in the bronchi. However, the studies cited by these researchers were conducted on mice and fetal lungs, so this observation cannot be clearly translated to the adult asthmatic patient population [72]. Furthermore, there is a single study in which children suffering from mild asthma who were not treated with corticosteroids had higher levels of TGF-β1 in plasma than children with a more severe course of asthma treated with corticosteroids [52]. Due to numerous inconsistencies between studies, it is currently impossible to clearly assess the interaction between corticosteroids and TGF in asthma. Studies on large groups of patients are necessary to assess the effect of corticosteroids on TGF levels and its function in asthma.

### 3.6. The Summary of TGF-β Role in Asthma

**Figure 4:**
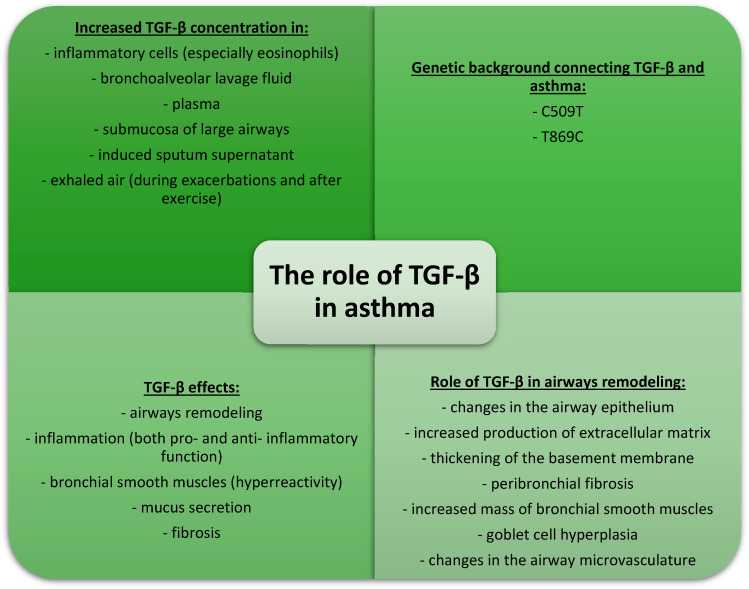
The role of TGF-β in asthma.

In conclusion, TGF-β plays an important role in airway remodeling in asthma. Its role in the induction of inflammation is twofold—both pro-inflammatory and anti-inflammatory. TGF-β2 appears to have an essential role in excessive mucus secretion in the respiratory tract. However, it should be noted that, similarly to data concerning COPD, many of the data come from studies on the mouse model of asthma. Thus, there is no certainty that the function of this cytokine in humans is precisely the same. The information about the role of TGF in asthma is summarized in Figure 4.

## 4. Putative Compounds Altering TGF-β Activity

Despite the effectiveness of traditional therapies in treating asthma and COPD, such as corticosteroid inhalations and bronchodilators, researchers and medical professionals are increasingly focusing on the search for alternative medications that can provide relief to patients affected by these conditions. Alternative treatments for asthma and COPD are becoming the subject of growing interest, and their development opens new perspectives for individuals struggling with these respiratory diseases.

Molecules that inhibit the action of TGF-β by targeting the extracellular components of the TGF-β signaling pathway comprise agents that prevent the activation of latent TGF-β, such as neutralizing anti-TGF-β antibodies, antisense oligonucleotides targeted at TGF-β isoforms, and decorin, an extracellular matrix protein that binds to TGF-β. Additional inhibitors include latency-associated protein (LAP), the soluble ectodomain of the TGF-β RIII (betaglycan), and a soluble TGF-β RII:Fc fusion protein [81]. Specific instances of these inhibitors are STX-100, an anti-integrin monoclonal antibody, and LSKL, a thrombospondin-1 peptide, both blocking the activation of latent TGF-β. Other examples include Fresolimumab (GC1008, with its mouse analog 1D11), a monoclonal pan-TGF-β ligand antibody; lerdelimumab (CAT-152), a monoclonal antibody specific to the TGF-β2 isoform; trabedersen, a TGF-β2 specific antisense oligonucleotide; and P144, a peptide based on the betaglycan ectodomain [81]. However, it is crucial to note that prolonged inhibition of TGF-β signaling, whether at the level of TGF-β isoforms or its receptors, may lead to severe adverse effects. As TGF-β regulates myriad intracellular signaling pathways to exert profibrotic effects, targeting those pathways offers an alternative strategy for potentially more specific pharmacologic intervention [82].

### 4.1. Natural Compounds

#### 4.1.1. Yu-Ping-Feng-San (YPFS)

Yu-Ping-Feng-San (YPFS) is a traditional Chinese medicine widely used to treat asthma in China [83]. Yang et al. conducted a study evaluating the effect of YPFS on treating COPD. The study used cigarette smoke and endotracheal lipopolysaccharide infusion to create a rat model of COPD. It was observed that TGF-β1 protein levels were significantly increased in COPD animals compared to normal rats, and that TGF-β1 expression was significantly reduced after YPFS administration. The anti-inflammatory effect of YPFS was achieved mainly through suppression of the TGF-β1/Smad2 signaling pathway, which may be involved in inhibiting inflammatory mediators and suppressing collagen deposition. Possible mechanisms may include Smad2 dephosphorylation, which may be responsible for the observed effects of YPFS on mitigating inflammation in vivo and in vitro [84]. The anti-inflammatory effects of YPFS in COPD were also confirmed in another similar study. The results demonstrate that YPFS significantly enhances oxidase activity while decreasing the levels of TNF-a, IL-6, TGF-β1, and phosphorylated-Smad2 (p-smad2) in YPFS-treated COPD rats compared to untreated COPD rats. Furthermore, the authors indicated that YPFS exerted anti-inflammatory effects in COPD rats by inhibiting the expression of inflammatory cytokines, potentially through the suppression of the TGF-β1/Smad2 signaling pathway [85].

#### 4.1.2. Berberine

A substance that has a potential of being used for treating diseases like COPD and asthma is berberine. Studies have already shown that this substance has anti-inflammatory properties and can significantly reduce airway inflammation, excessive mucus secretion, or increased expression of P38 MAPK and ERK in mice [86,87]. In a study from 2019, it was observed that high doses of berberine reduced the expression of TGF-β1, Smad2, and Smad3 in the cigarette smoke extract (CSE)-induced COPD mice model. One may speculate that pretreatment with berberine can attenuate CSE-induced airway inflammation in mice, in which TGF-β1/Smads signaling may be involved [88]. In a recently published paper, a problem of berberine’s poor permeability was described that might potentially hinder its utility in treating asthma and/or COPD. Berberine was encapsulated in monoolein-based liquid crystal nanoparticles (BM-LCN), and its potential to inhibit TGF-β-induced remodeling features in human bronchial epithelial cells was investigated. The substance appeared to significantly reduce the levels of proteins up-regulated by TGF-β and increase the levels of proteins that are downregulated by TGF-β. Hence, BM-LCN can potentially be of use in chronic respiratory diseases mentioned above [89].

#### 4.1.3. Betalains

Betalains are water-soluble plant pigments, and their primary source of occurrence is plants of the order *Caryophyllales*. Preclinical studies have shown that betanin exhibits antioxidant, anti-inflammatory, hepatoprotective, anticancer, antidiabetic, antilipidemic, antimicrobial, radioprotective, and antiproliferative activities [90]. In a study from 2021, the authors tested the therapeutic potential of betalain against ovalbumin (OVA)-induced asthma in a mouse model by inhibiting the TGF-β1/Smad signaling pathway. The results show betalain’s anti-inflammatory effects and reduced IgE, eotaxin, and cytokine production. Nitric oxide levels and oxidative stress were also reduced, and lung mechanics improved. The drug significantly decreased gene expression of TGF-β and its downstream signaling protein Smad. Further studies on betalain are necessary to prove its utility in treatment of asthma in humans [91].

#### 4.1.4. Osthole

Another putative anti-asthmatic compound is osthole, a natural product found in several medicinal plants, such as *Cnidium monnieri* and *Angelica pubescens*. The studies have shown that osthole exhibited neuroprotective, osteogenic, immunomodulatory, anticancer, hepatoprotective, cardiovascular, and antimicrobial activities [92]. Osthole has been investigated in an OVA-induced asthma mouse model. Osthole effectively reduces lung inflammation induced by ovalbumin through the inhibition of IL-33/ST2 signaling pathways in asthmatic mice [93]. In another study, osthole application significantly inhibited TGF-β1-induced apoptosis of human bronchial epithelial 16HBE cells. Moreover, the promoter effect of TGF-β1 on the proliferation of human bronchial epithelial cells was reversed. Inhibition of TGF-β1-induced activation of the Smad2/3 pathway and MAPKs, as well as amelioration of epithelial damage and subepithelial fibrosis in the airways in a mouse model of asthma, were also confirmed [94].

#### 4.1.5. Nerolidol

Nerolidol is a naturally occurring sesquiterpene alcohol with a wide range of applications [95]. One of its features is anti-asthmatic activity. Mice sensitized with OVA were given various doses of nerolidol. It appeared to reduce inflammatory cell infiltration, cup cell number, lung collagen deposition, and OVA-specific IgE levels in serum and alveolar fluid of asthmatic mice. It also caused thinning of the bronchial basement membrane in mice with asthma. Airway smooth muscle cell (ASMC) hyperplasia is an essential feature of airway remodeling in asthma. These cells were injected with 10 ng/mL TGF-β to mimic the pathological environment in asthma. Nerolidol has been shown to exhibit inhibitory effects on the TGF-β/Smad signaling pathway in both OVA-induced mouse lung tissue and TGF-β-stimulated airway smooth muscle cells [96]. This observation makes nerolidol a potential candidate for the treatment of asthma.

#### 4.1.6. Diosmetin

Diosmetin is an anti-asthmatic drug with anti-inflammatory properties which has a potential to significantly decrease TGF-β, MMP-9, and VEGF levels [9,97]. Epithelial–mesenchymal transition (EMT) is a pivotal repairing and airways remodeling process, particularly in respiratory diseases like asthma. TGF-β1 promotes EMT and the generation of reactive oxygen species (ROS) in human bronchial epithelial (HBE) cells. Diosmetin has demonstrated its ability to prevent TGF-β1-induced intracellular ROS generation. Moreover, diosmetin significantly inhibits the TGF-β1-induced PI3K/Akt and MAPK pathways in human bronchial epithelial cells (HBE) cells [98]. It was also shown that administration of high doses of diosmetin (0.5 mg/kg) significantly reduced the total counts of eosinophils and neutrophils [9].

#### 4.1.7. Amygdalin

Amygdalin is a cyanogenic diglucoside, a natural compound well-known for its anti-inflammatory, anti-fibrotic, and immunoregulatory potential. It is found in certain seeds, particularly in apricot seeds [99,100,101]. Amygdalin administration to interstitial fibroblasts in culture reduces their proliferative capacity and alters their ability to secrete TGF-β1 [102]. Treatment with this compound alleviated airway wall remodeling and reduced the expressions of epithelial–mesenchymal transition markers in mouse asthmatic model. In bronchial epithelial cells treated with TGF-β, amygdalin treatment decreased levels of α-SMA, vimentin, and fibronectin without compromising cell viability [103]. Wang et al. investigated the EMT process in human bronchial epithelial cells and mice exposed to cigarette smoke administered with various concentrations of amygdalin. It was revealed that E-cadherin expression was elevated and the levels of vimentin, TGF-β1, and phosphorylated Smad2/3 were reduced in the groups subjected to amygdalin, as compared to the control. This research provides evidence supporting the protective role of amygdalin in murine EMT during COPD. It was also shown that amygdalin at a dose of 20 mg/kg/d demonstrated a significant increase in FEV in mice receiving amygdalin, compared with the control group. It was also shown that amygdalin treatment had a protective effect on long-term CSE-induced deterioration of spirometry parameters [104].

#### 4.1.8. Epigallocatechin Gallate (EGCG)

Epigallocatechin gallate (EGCG) is a monomer of tea polyphenols of proven high antioxidant and DNA-protective activity [105,106]. EGCG has also potential to suppress the secretion of some cytokines, including TGF-β1 [107]. At a dosage of 20 mg/kg, EGCG significantly alleviated asthmatic symptoms, reduced lung inflammatory cell infiltration, and decreased the levels of inflammatory cytokines, such as interleukin (IL)-2, IL-6, and tumor necrosis factor (TNF)-α. EGCG also mitigated airway inflammation in asthmatic mice, leading to a decrease in Th17 cells percentage and an increase in the percentage of Treg cells. The anti-inflammatory effects of EGCG are accomplished through modulation of the TGF-β1 signaling pathway [106].

#### 4.1.9. Aloin

Aloin is an anthraquinone compound commonly present in the aloe vera plant. Anthraquinones, including aloin A and B, demonstrate antiviral, antimicrobial, and anti-inflammatory properties [108,109]. In both in vitro cell experiments and in vivo animal studies, aloin exhibited the ability of diminishing fibrosis by modulating the TGF-β/Smad2/3 signaling pathway. Additionally, aloin alleviated TGF-β1-induced inflammation. Some studies suggest a potential therapeutic role for aloin in addressing fibrosis-related conditions [110,111].

Furthermore, in a murine asthma model induced by OVA treatment, total counts of neutrophils, eosinophils, and macrophages were observed, as well as significant increases in concentrations of interleukins IL-4, IL-5, and IL-13. However, the administration of aloin mitigated these effects. Overall, aloin treatment ameliorated airway hyperresponsiveness, airway remodeling, inflammation, and oxidative stress in OVA-treated mice [112].

It is also demonstrated that aloin has a protective effect in combined allergic rhinitis and asthma syndrome (CARAS). The medium and high aloin doses (20 and 40 mg/kg, respectively) caused reduced eosinophil infiltration compared to the placebo group in mice [113].

#### 4.1.10. Quercetin

Quercetin (3,3′,4,5,7-pentahydroxyflavone) is a natural polyphenol flavonoid occurring in some fruits and vegetables [114]. Quercetin suppresses TGF-β-induced responses via inhibition of the Akt/mammalian target of the rapamycin (mTOR) pathway and suppression of fibrotic factors such as collagen I, collagen III, and IL-6 [115,116,117]. It has shown substantial efficacy and the potential to alleviate major asthma symptoms, including eosinophil and neutrophil recruitment, activation of bronchial epithelial cells, collagen and mucus production, and airway hyperactivity [118]. In the study by Rajizadeh et al., asthmatic rats were administered intraperitoneally daily for a week with quercetin (50 mg/kg) and dexamethasone (2.5 mg/kg). The results demonstrated that quercetin reduced the expression of Gata-3, TNF-α, TGF-β1, IL-1β, and α-SMA genes. Additionally, after asthma treatment, quercetin decreased IL-6 and TNF-α levels while increasing IL-10 levels in lung tissue. Hence, quercetin effectively mitigated oxidative stress and inflammation caused by asthma [119]. In the study by McCluskey and colleagues, COPD basal cells treated with quercetin exhibited increased transepithelial electrical resistance (TER) and regeneration of the airway epithelium. This regeneration was characterized by an augmentation in ciliated cells and a reduction in goblet cells and IL-8. Quercetin also upregulated genes associated with tissue and epithelial development and differentiation [120].

#### 4.1.11. Kaempferol

Nicotinamide adenine dinucleotide phosphate oxidase 4 (NOX4) downregulation was found to inactivate the TGF-β1-Smad2/3 pathway in mice with asthma. Diphenyleneiodonium (DPI) 0.5 mg/kg, NOX4 inhibitor, was intraperitoneally injected into mice. As a result, airway remodeling and inflammation were alleviated [121]. Although the exact signaling associations between NOX4 and TGF-β1 remain unknown, NOX4 contributed to ROS formation, which enhanced TGF-β1-induced proliferation and hypertrophy of human airway smooth muscle [122]. Future studies are needed to evaluate the efficacy and safety of NOX4 blocking in the treatment of asthma. Kaempferol (3,4′,5,7-tetrahydroxyflavone) is a natural compound found in apples, grapes, tomatoes, green tea, broccoli, pine, and ginkgo leaves [123,124]. An in vitro study on human bronchial epithelial cells (BEAS-2B) demonstrated that kaempferol treatment decreases NOX4 expression. Airway inflammation and remodeling were reduced through attenuating NOX-4-mediated autophagy [125].

### 4.2. Synthetic Compounds

#### 4.2.1. Nintedanib

Currently used to treat idiopathic pulmonary fibrosis, nintedanib is drug worthy of consideration in asthma treatment [126]. Studies have shown its anti-inflammatory effects in a model of chronic asthma [127]. Lee et al. examined the impact of oral nintedanib on airway hyperresponsiveness and smooth muscle cells using a mouse model of experimental asthma. The results confirm that nintedanib treatment, among other beneficial effects, significantly reduces elevated TGF-β levels in the mouse asthma model, with no significant difference compared to dexamethasone [127]. In a study focusing on the anti-fibrotic effects of nintedanib, its inhibitory effects on TGF-β signaling, specifically TGF-β type II receptor tyrosine phosphorylation, Smad3 activation, and p38 mitogen-activated protein kinase, were demonstrated [126]. This study also confirmed the mechanism of TGF-β downregulation in the asthma model.

#### 4.2.2. Tranilast

Tranilast (N-[3′,4′-dimethoxycinnamoyl]-anthranilic acid) is an analog of a tryptophan metabolite. Its mechanism of action relies on histamine release inhibition, one of the most important mediators of allergic reactions [128,129]. It was also observed to suppress the production of nitric oxide, prostaglandin E2, TNF-α, and IL-1b in macrophage cells upon lipopolysaccharide stimulation, as well as to inhibit hypersensitivity reactions mediated by mast cells [130].

One of the anti-fibrotic effects of tranilast is caused by the inhibition of TGF-β1, a factor known to enhance collagen synthesis [131,132,133]. Tranilast is a generally well-tolerated drug with a good safety profile for the treatment of asthma with low unspecific reaction rates and toxicity. Consequently, it is a safe and proven drug with minimal long-term side effects [129]. The conventional dose of tranilast for bronchial asthma, allergic rhinitis, atopic dermatitis, keloid, and hypertrophic scars is 300 mg/day. Higher doses of the drug used in a large clinical trial caused elevation of liver enzymes in approximate 10% of the study group [134,135]. Long-term tranilast administration suppressed bronchial hypersensitivity in asthmatics. It was also shown to significantly decrease eosinophil count and the level of IgE specific to *Dermatophagoides farina* in asthmatic children [129,136]. Tranilast was also proved to protect against acute respiratory distress syndrome and early pulmonary fibrosis in vivo in rats. Furthermore, tranilast promoted the proliferation of type II alveolar epithelial cells and pulmonary microvascular endothelial cells while inhibiting the proliferation of pulmonary fibroblasts of rats in vivo [137].

Exacerbations of asthma or COPD associated with respiratory viral infection may resist the anti-inflammatory actions of glucocorticosteroids (GCs) [138]. Xia and colleagues demonstrated that tranilast inhibited respiratory syncytial virus (RSV) infection-induced mRNA expression of TGF-β1 and plasminogen activator inhibitor-1 (PAI-1). The researchers found that pretreatment of epithelial cells with tranilast reduced the expression and activity of TGF-β and restored GC sensitivity. Hence, tranilast effectively mitigated GC insensitivity during RSV infection-induced bronchiolitis or exacerbations of asthma/COPD [139].

Further research on the effectiveness of tranilast in viral infections could support the use of TGF-β modulators as a potential approach for preventing or treating GC insensitivity during these respiratory conditions [139].

#### 4.2.3. Pan-PDE Inhibitors

Phosphodiesterase (PDE) inhibitors facilitate the increase in both cAMP and cGMP levels, resulting in the relaxation of airway smooth muscle, bronchodilation, and the inhibition of specific inflammatory pathways via modulation of T-cell activation and proliferation [140,141]. Pan-PDE inhibitors can inhibit various isoforms of PDEs.

Recently, a novel class of pan-PDE inhibitors, comprising 7,8-disubstituted purine-2,6-dione derivatives, has been synthesized. Among them, a 145-aa pan-PDE inhibitor demonstrated significant efficacy in curtailing the FMT process, and in inhibiting proliferation, migration, and contraction. The robust anti-remodeling effects of 145-aa pan-PDE inhibitor were dependent on activating the cAMP/protein kinase A pathway, leading to the inhibition of TGF-β1 secretion. These findings suggest that the TGF-β pathway is a critical target for PDE inhibitors, resulting in inhibitory effects on cellular responses implicated in airway remodeling [142].

The inhaled pan-PDE inhibitors significantly decreased airway inflammatory cell infiltration, eosinophil recruitment, Th2 cytokine levels in bronchoalveolar lavage fluid, and total and specific IgE levels in plasma in asthma patients [143]. Unlike the selective PDE4 inhibitors, like roflumilast or cilomilast, pan-PDE inhibitors may offer superior inhibition of TGF-β1-induced airway smooth muscle cell remodeling [144].

Utilizing the above knowledge may help improve the clinical condition of patients with asthma, but further research is needed on the effect of TGF-β inhibition in asthma (Table 1).

## 5. Summary

Undoubtedly, TGF-β plays an essential role in the pathogenesis of both COPD and asthma. Most studies indicate that TGF expression is increased in both of these diseases. In many processes occurring in both diseases, TGF-β has a similar function: it promotes the remodeling of the airways by increasing the deposition of extracellular matrix, inducing fibrosis, thickening of the basal membrane, altering vascularization, and influencing the production of MMPs and their inhibitors (TIMP) displaying both pro- and anti-inflammatory roles. These changes may lead to irreversible obstructions in both diseases. Additionally, TGF-β influences the development of emphysema in COPD patients, but its role in this process is still unclear.

There are also several differences in how TGF-β affects specific aspects of COPD and asthma. Firstly, in the case of asthma, most researchers agree on the dual function of TGF-β in inflammation. However, in the case of COPD, the researchers more often emphasize the anti-inflammatory role of TGF-β. Secondly, there are inconsistences in TGF-β2 in asthma, while TGF-β is believed to decrease the mucus production in COPD [51,145]. The comparison of the role of TGF-β in COPD and asthma is presented in Table 2.

## 6. Conclusions

The preclinical testing results of drugs targeting non-canonical TGF-β signaling pathways in experimental models of fibrosis are promising. However, because these results have not been translated into meaningful anti-fibrotic therapies in a clinical application, the fundamental fibrotic mechanisms in mice and humans may be equivocal. As a result, more experimental systems are needed to predict outcomes in human trials. The interactions between Smad-dependent and non-Smad pathways in facilitating the fibrogenic effects of TGF-β are not thoroughly elucidated and remain an area that necessitates further investigation.

## Figures and Tables

**Table 1:** Drugs altering TGF-β activity in asthma and COPD.

Drug	Disease	Mechanism of Action on TGF-β	Dose	Response to Treatment	Ref.
Yu-Ping-Feng-San (YPFS)	COPD	suppression of the TGF-β1/Smad2 signaling pathway	0.5 g/kg/day	anti-inflammatory effect—reduction in TGF-β1 expression, suppressed release of pro-inflammatory cytokines, and collagen deposition	[84,85]
Berberine	COPD, asthma	TGF-β1/Smads signaling might be involved	25 mg/kg	attenuation of CSE-induced airway inflammation, reduction in TGF-β1, Smad2, and Smad3	[88]
Nintedanib	asthma	need more research	0.2 mL of PBS containing nintedanib (50 or 100 mg/kg)	reduction in TGF-β levels, suppression of DGFRß,VEGFR2, and FGFR3; reduction in eosinophilic airway inflammation and the remodeling process	[127]
Betalains	asthma	inhibiting the TGF-β1/Smad signaling pathway	25 mg/kg or 50 mg/kg	reduction in TGF-β gene expression and its downstream signaling protein Smad; anti-inflammatory effect; reduction in oxidative stress, production of IgE, eotaxin, cytokines, lower nitric oxide levels, and improvement in lung mechanics	[91]
Osthole	asthma	inhibition of TGF-β1-induced activation of the Smad2/3 pathway and MAPKs	50 mg/kg	inhibits TGF-β1-induced apoptosis of human bronchial epithelial cells, amelioration of epithelial damage and subepithelial fibrosis	[94]
Nerolidol	asthma	inhibitory effect on the TFG-β/Smad signaling pathway	ND	reduction in TGF-β levels, reduction in inflammatory cell infiltration, cup cell number, lung collagen deposition, and OVA-specific IgE levels	[96]
Tranilast	asthma/COPD	inhibiting TGF-β-induced protein kinase phosphorylation	300 mg/day	suppressed bronchial hypersensitivity in asthmatics, decreased eosinophil counts and specific IgE, reduced the expression and activity of TGF-β, restored GC sensitivity	[129,134,136,139]
Diosmetin	asthma	inhibiting TGF-β1-induced phosphorylation of PI3K/Akt and MAPK	0.5 mg/kg	reduction in the counts of total cells, eosinophils, and neutrophils	[9,98]
Pan-PDE inhibitors	asthma	activation of the cAMP/protein kinase A/cAMP response element-binding protein pathway, leading to the inhibition of TGF-β	ND	decreased airway inflammatory cell infiltration, eosinophil recruitment, IgE, and Th2 cytokine levels	[142,143]
Amygdalin	COPD	inhibitory effect on the TFG-β/Smad signaling pathway	20 mg/kg/d	decreased levels of TGF-β1, α-SMA, vimentin, and fibronectin increase FEV	[102,104]
Epigallocatechin gallate (EGCG)	asthma	decrease the expression of TGF-β1 and phosphorylated (p)-Smad2/3	20 mg/kg	alleviated asthmatic symptoms, reduced lung inflammatory cell infiltration, decreased the levels of IL-2, IL-6, TNF-α, and Th17 cells, and increased the percentage of Treg cells	[106,107]
Aloin	asthma/CARAS	inhibitory effect on the TFG-β/Smad signaling pathway	20, 40 mg/kg	decrease neutrophils, eosinophils, macrophages, and interleukins (IL)-4, IL-5, and IL-13	[107,109]
Quercetin	asthma/COPD	suppresses TGF-β-induced responses; it inhibits the Akt/mTOR, reduces collagen I, collagen III, and IL-6	50 mg/kg and dexamethasone (2.5 mg/kg) intraperitoneally for a week	reduced the expression of Gata-3, TNF-α, TGF-β1, IL-1β, and α-SMA genes, decreased IL-6 and TNF-α levels while increasing IL-10 levels	[115,116,119]
Kaempferol	asthma	reducing NOX4 expression results in the inactivation of the TGF-β1-Smad2/3 pathway	ND	reduce airway inflammation and remodeling	[111,114]

**Table 2:** The comparison of the role of TGF-β in COPD and asthma.

	Role of TGF-β in COPD	Role of TGF-β in Asthma
**TGF concentration**	↑	↑
**Airways remodeling**	↑	↑
**Production of extracellular matrix**	↑	↑
**Fibrotic changes**	↑	↑
**Thickening of basal membrane**	↑	↑
**Vascular changes**	↑	↑
**Production of MMPs**	↑	↑/↓
**Pro- (** **↑** **) and anti- (** **↓** **) inflammatory function**	↑/↓↓↓	↑/↓
**Development of emphysema**	↑	↓
**Mucus secretion**	↓	↑/↓
